# Association between Dietary Antioxidant Capacity in Midlife and Depressive Symptoms in Late Life: The Singapore Chinese Health Study

**DOI:** 10.3390/antiox13050576

**Published:** 2024-05-08

**Authors:** Huiqi Li, Li-Ting Sheng, Bee Choo Tai, An Pan, Woon-Puay Koh

**Affiliations:** 1 Healthy Longevity Translational Research Programme, Yong Loo Lin School of Medicine, National University of Singapore, Singapore 117597, Singapore; huiqi_li@u.nus.edu; 2 Phase I Clinical Trial Center, The Affiliated Suzhou Hospital of Nanjing Medical University, Suzhou Municipal Hospital, Gusu School, Nanjing Medical University, Suzhou 215000, China; 3 Drug Clinical Trial Center, Peking University Third Hospital, Beijing 100191, China; 4 Saw Swee Hock School of Public Health, National University of Singapore, Singapore 117549, Singapore; 5 Department of Epidemiology and Biostatistics, Ministry of Education Key Laboratory of Environment and Health, School of Public Health, Tongji Medical College, Huazhong University of Science and Technology, Wuhan 430030, China; 6 Singapore Institute for Clinical Sciences, Agency for Science Technology and Research (A*STAR), Singapore 138632, Singapore

**Keywords:** antioxidants, vitamins, depression, older adults, carotenoids

## Abstract

Preclinical and limited epidemiological studies suggest that oxidative stress may be implicated in geriatric depression. Our study investigated the association between midlife dietary total antioxidant capacity (TAC) and depressive symptoms in late life among 13,712 participants in a population-based cohort of Chinese in Singapore. At baseline (1993–1998), intake of antioxidants from diet and supplements at a mean age of 52.4 years was estimated using a validated food frequency questionnaire to derive two dietary TAC indices from vitamins C and E, carotenoids and flavonoids: the Comprehensive Dietary Antioxidant Index (CDAI) and Vitamin C Equivalent Antioxidant Capacity (VCEAC). At follow-up 3 (2014–2016), when participants were at a mean age of 72.5 years, depressive symptoms were assessed using the Geriatric Depression Scale, and depression, defined as having ≥5 symptoms, was presented in 3173 (23.1%) participants. Both CDAI and VCEAC indices were inversely associated with odds of depressive symptoms in a stepwise manner: the OR (95% CI) comparing the extreme quartiles was 0.73 (0.64–0.83; *P*_trend_ < 0.01) for the CDAI and 0.77 (0.68–0.87; *P*_trend_ < 0.01) for the VCEAC. Specifically, higher intakes of vitamin C, carotenoids, and flavonoids were associated with a lower likelihood of depressive symptoms. Our findings support the recommendation of an antioxidant-rich diet for the prevention of depression.

## 1. Introduction

Older adults have an increased risk of developing depressive symptoms that can lead to reduced psychosocial functioning and quality of life, increased cognitive decline, and premature mortality, which, in turn, result in considerable individual and societal health burdens [1]. Although pharmacological treatment has been proven to be effective in relieving some serious symptoms [2], older adults often respond less favorably to drug therapies due to slower metabolism and comorbidities, resulting in frequent relapses and potentially more severe consequences [3]. As such, identifying modifiable factors at an early stage to prevent depression or delay its progression with aging is a major public health priority, especially in the era when population aging is of growing concern worldwide [4]. 

Although the pathogenesis of geriatric depression has not been fully understood, oxidative stress and neuroinflammation are two established pathways underlying the development of depression in aging [5,6]. Oxidative stress is generated when the balance between reactive oxygen species (ROS) and antioxidant defense systems is markedly shifted towards oxidative potentials [5]. Experimental studies have indicated that excessive ROS in the brain can directly cause neuroinflammation, disruption of neural signaling, and even neuronal death, which are implicated in neurodegenerative diseases [7,8]. Epidemiological studies among humans have also shown elevated biomarkers of oxidative stress in patients with late-life depression [9]. 

As oxidative stress is a key to brain health, counteracting the overproduction of ROS holds potential in the prevention of late-life depression. In support of this, accumulating evidence has shown that various antioxidants derived from diet can enhance the body’s antioxidant defenses and combat oxidative stress by boosting plasma antioxidant levels, thereby reducing the risk of late-life depression [10,11]. However, most of the previous studies on the association between dietary antioxidants (i.e., vitamin C, vitamin E, carotenoids, and flavonoids) and late-life depression focused on individual antioxidants [12,13,14], which could not reflect the total antioxidant power of dietary intake. In addition, many antioxidant nutrients share common food sources and are highly correlated with each other. Hence, it is challenging to disentangle their independent effects with accuracy unless efforts are made to account for their cumulative and synergistic effects. 

To overcome the aforementioned challenges, recently, several cross-sectional studies have used the total antioxidant capacity (TAC) from dietary intake to assess the synergistic networking of different antioxidants from diverse food groups and to explore the association of dietary TAC with late-life depression. However, findings from current studies have remained controversial [15,16,17]. Two small cross-sectional studies demonstrated an inverse association between dietary TAC, assessed by the ferric reducing antioxidant power (FRAP) and oxygen radical absorbance capacity (ORAC), and late-life depression among peri-and postmenopausal women [15,16]. Notably, the evaluation of TAC using FRAP and ORAC could have limitations as these two indices were based on different underlying mechanisms that used specific radical or oxidant sources to assess the antioxidant capacity of food items [18,19]. Another cross-sectional study among climacteric women, however, did not find any differences in dietary TAC reflected by equivalents of vitamin C between depressed and non-depressed individuals [17]. To the best of our knowledge, no prospective study has ever evaluated the cumulative effect of dietary antioxidants in relation to late-life depression in the general older population.

Therefore, the current study aimed to evaluate the association of dietary TAC and its individual components in midlife with depressive symptoms in late life after about 20 years of follow-up in the Singapore Chinese Health Study, which is an ongoing population-based prospective cohort study. We hypothesized that after accounting for participants’ demographic characteristics, lifestyle factors, history of medical conditions, usual diet, and aging-related physical or psychosocial factors, a higher level of dietary TAC in midlife would be associated with a lower likelihood of depression in late life.

## 2. Materials and Methods

### 2.1. Study Design

We used data from the Singapore Chinese Health Study, which was established between April 1993 and December 1998, by enrolling 63,257 participants of Chinese ethnicity (27,954 men and 35,303 women) who were aged 45–74 years [20]. Specifically, all participants were Singaporean citizens or permanent residents living in public housing estates (where 86% of the Singapore population resided during the period of recruitment) and either of the Hokkien or Cantonese dialect group, who originated from Fujian and Guangdong provinces in southeast China, respectively. After enrollment, the participants were followed up every 5–6 years, either through phone or in-person interviews, to update their information on lifestyle factors and medical conditions. During the follow-up 3 interviews conducted through home visits between July 2014 and February 2016, aging outcomes, including depressive symptoms, cognitive status, physical well-being, and functional ability, were assessed through in-person interviews using standard questionnaire instruments. Due to funding restraints, the follow-up 3 visits were stopped prematurely, and a total of 17,107 surviving participants who were aged 61–96 years were successfully re-contacted. 

The study was conducted according to the guidelines of the Declaration of Helsinki and approved by the Institute Review Board of the National University of Singapore (NUS-IRB Reference Code: L04-026). All participants were informed about the aims of the study and provided written informed consent.

### 2.2. Dietary Exposure Assessment

At baseline, trained interviewers administered a semi-quantitative food frequency questionnaire (FFQ) to collect data on the intake frequency and portion size of 165 food items and dishes over the past year for individuals [20]. The use of supplements was also collected for selected micronutrients that included vitamin C, vitamin E and β-carotene. The FFQ was subsequently validated against two 24-h dietary recalls that were administered on one weekday and one weekend day among 810 participants randomly chosen from the cohort. The daily intake of nutrients and total energy were calculated for each participant using the Singapore Food Composition Database [20], which was specifically developed for this cohort. The paired values of mean energy intake and consumption of selected macro- and micro-nutrients estimated using the 24-h dietary recalls and the FFQ were comparable, as they were within 10% difference of each other’s values. Furthermore, the correlation coefficients for the intake levels of selected vitamins from 24-h dietary recalls versus FFQ ranged from 0.36–0.67 [20]. 

A total of 12 antioxidants from food and supplement sources were included in the analyses: carotenoids (including α-carotene, β-carotene, β-cryptoxanthin, lycopene, and lutein), flavonoids (including anthocyanins, flavan-3-ols, flavanones, flavones, and flavonols), vitamin C, and vitamin E. We used two previously established methods to construct dietary TAC indices: the Comprehensive Dietary Antioxidant Index (CDAI) and Vitamin C Equivalent Antioxidant Capacity (VCEAC). The CDAI was calculated based on the modeling developed by Wright et al. [21]. Briefly, the intake levels of 12 antioxidants were first standardized by subtraction of the corresponding mean value and division by the standard deviation. After that, given that the nutrient subclasses in each carotenoid and flavonoid group were structurally and functionally correlated, principal component analyses were conducted separately for these two groups to obtain the first principal components. Finally, the standardized intake levels of the first principal components of the carotenoid and flavonoid groups, vitamin C and vitamin E, were summed to obtain the CDAI index. On the other hand, we calculated the VCEAC index based on the database of vitamin C equivalents (VCEs) by Floegel et al. [22]. The intake levels of the 12 antioxidants were multiplied by their corresponding VCE values, and then all the values were summed to obtain the total VCEAC. These two dietary TAC indices and their individual components were adjusted for daily energy intake using the residual method [23].

### 2.3. Covariates Assessment

All covariates included in the analyses were collected using structured questionnaires administered by trained interviewers at baseline and follow-up 3. The baseline questionnaire included information on demographics, height, weight, usual diet habits, lifestyles (i.e., smoking status, alcohol intake, and physical activity), and self-reported history of physician-diagnosed medical conditions (i.e., diabetes, hypertension, and cardiovascular diseases). Body mass index was calculated by the formula: weight (kg)/height (m^2^). Participants who spent less than 30 min per week on moderate activity, vigorous activity or strenuous sports were considered physically inactive. 

During follow-up 3, we measured aging-related factors, including instrumental limitations, self-rated health, and social activity, which were previously reported to be related to late-life depression [24,25]. Specifically, self-rated health was assessed by asking participants a single question: “In general, would you say your health is: excellent, very good, good, fair, or poor?”. Independent living was measured by the Lawton instrumental activities of daily living scale (IADL) [26], and those with at least one limitation were considered to have instrumental limitations. We measured social activity by asking participants the weekly hours they spent in a social or workgroup, church-connected group, self-help group, charity, public service, or community group. Respondents who participated < 1 h/week were considered as having no social activity.

### 2.4. Depressive Symptoms Assessment

We used the 15-item Geriatric Depression Scale (GDS-15) as a screening tool to evaluate depressive symptoms for each participant at follow-up 3 interviews when the surviving participants were aged 61–96 years. This scale had previously been validated among community-dwelling older adults living in Singapore, and those with a GDS-15 score ≥ 5 were considered to be depressed [27].

### 2.5. Statistical Analysis

**Figure 1:**
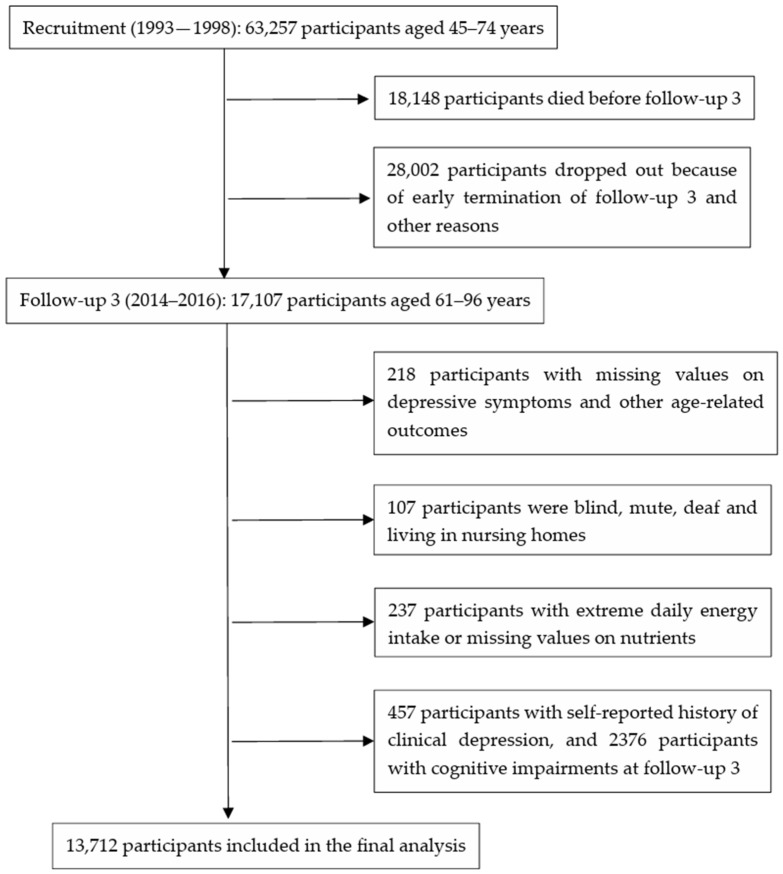
Flow chart depicting the inclusion of participants in the analyses.

We excluded 218 participants with missing values on depressive symptoms and other aging-related factors, 107 participants who were blind, speech-impaired or deaf, 237 participants with extreme energy intake (<600 or >3000 kcal/day for women and <700 or >3700 kcal/day for men) or missing values on antioxidants intake, 2376 participants with cognitive impairment measured by Singapore-modified Mini-Mental State Examination and 457 participants with self-reported history of clinical depression at follow-up 3, leaving 13,712 participants in the current analyses (Figure 1). The characteristics of participants at baseline or follow-up 3 were compared by depressed status measured at follow-up 3 and by quartiles of CDAI and VCEAC. The chi-square test was used for the comparison of categorical variables. Student’s t-test or the Mann–Whitney U test was used for the comparison of continuous variables by depressed status, and analysis of variance was used for the comparison of continuous variables by quartiles of CDAI and VCEAC. Multivariable logistic regression models were used to calculate odds ratios (ORs) and 95% confidence intervals (CIs) for the associations between quartile levels of the two TAC indices and late-life depressive symptoms, using the lowest quartiles as the referent groups. We also repeated the analyses for major components that defined the two TAC indices. The linear trend was tested by including the median values of the quartiles as a continuous variable in the models.

We adjusted for the following covariates in model 1: age (years) and marital status (married, non-married) at follow-up 3, sex, level of education (no formal education, primary school education, or secondary school or higher), dialect group (Hokkien or Cantonese) and baseline daily energy intake (kcal/d). In model 2, we further adjusted for baseline smoking status (never, former, or current smokers), alcohol intake (never/monthly, weekly, or daily), physical activity level (<0.5, 0.5–3.9, or ≥4.0 h of moderate or vigorous activities per week), BMI (<18.5, 18.5–22.9, 23.0–27.4, or ≥27.5 kg/m^2^), sleep duration (≤5, 6–8, or ≥9 h per day), weekly supplement use (yes or no), and baseline medical history of hypertension, diabetes, cardiovascular diseases (yes or no). In model 3, we additionally adjusted for instrumental limitations (0 or ≥1 limitations), self-rated health (good, fair, or poor) and social activity (yes or no) at follow-up 3. 

We conducted three sensitivity analyses to test the robustness of our study: (i) we excluded those with diabetes, hypertension, and cardiovascular diseases at baseline (n = 3078), as previous studies have reported that these comorbidities could be risk factors of late-life depression [28]; (ii) since we were studying aging-related depressive symptoms, we excluded those who were aged ≥ 65 years at baseline (n = 535); (iii) we applied inverse probability weighted regression models that could partially account for potential selection bias due to loss-to-follow-up during follow-up 3 visits (Supplemental methods). 

Effect modification was tested by including a cross-product term between quartiles of dietary TAC and the following potential modifiers in the model: age at GDS measurement (<70 or ≥70 years), sex, baseline BMI (<23 or ≥23 kg/m^2^), smoking status (non-smoker or ever smoker), and baseline history of chronic diseases (yes or no). The inverse probability weighted regression analysis was performed using STATA/MP version 14.0, and all the other analyses were conducted by SAS version 9.4 (SAS Institute). Statistical significance was defined as 2-sided *p* values < 0.05.

## 3. Results

The participants were aged 52.4 ± 5.9 years at baseline and 72.5 ± 6.1 years at GDS measurement during follow-up 3. Women accounted for 58.1% of the participants in this study. Participants who had depressive symptoms measured by GDS at follow-up 3 were more likely to be women, have lower educational levels, smoke, and report chronic diseases at baseline, as well as poor physical health and less social engagement at follow-up 3. Additionally, they consumed lower levels of antioxidant nutrients from plant-based foods at baseline compared to those without depressive symptoms (Table 1). Characteristics of participants at baseline and follow-up 3 across quartiles of dietary TAC were shown in Table 2. Participants in higher quartiles of dietary TAC were more likely to be women and have higher educational levels and BMI at baseline but less likely to be smokers or daily drinkers. Additionally, they were more likely to be functionally independent and to report good self-rated health and social engagement at follow-up 3. 

After a mean follow-up of 19.6 years, 3173 (23.1%) participants were considered to have depressive symptoms at follow-up 3. After accounting for demographics, lifestyle factors, medical conditions, supplement use, and other aging-related factors, higher quartiles of both CDAI and VCEAC indices were associated with a lower likelihood of late-life depressive symptoms. Comparing the extreme quartiles, the OR (95% CI) for late-life depressive symptoms was 0.73 (0.64–0.83) for CDAI and 0.77 (0.68–0.87) for VCEAC, respectively (all *P*_trend_ < 0.01, Table 3). The results were robust and remained essentially unchanged in sensitivity analyses that excluded those with pre-existing chronic diseases and those aged ≥ 65 years at baseline, as well as when applying inverse probability weighted regression models that addressed the potential influence of selection bias (Supplementary Tables S2–S4). We did not observe any significant interaction between the two dietary TAC indices and age at GDS measurement, sex, baseline BMI, baseline smoking status and baseline history of chronic diseases for their associations with late-life depressive symptoms (all *P* interaction ≥ 0.23, Supplement Table S1).

**Figure 2:**
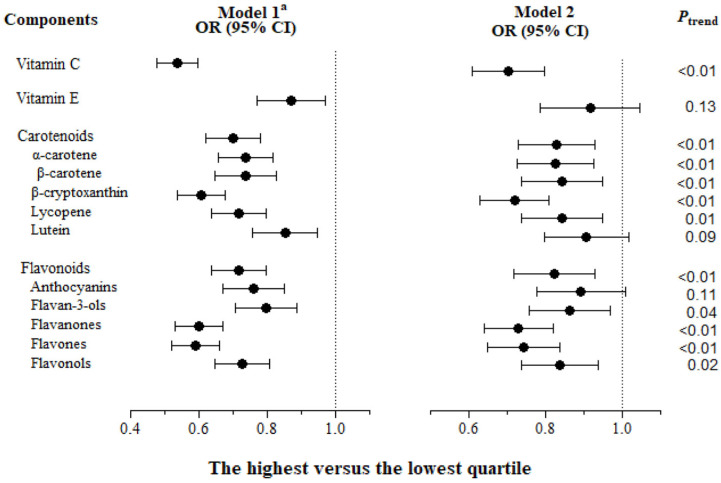
Unadjusted and multivariable-adjusted ORs (95% CIs) of depressive symptoms for energy-adjusted daily intake of individual antioxidants in dietary total antioxidant capacity (n = 13,712). Model 1 was unadjusted; model 2 was adjusted for age at the third follow-up, sex, dialect group, level of education, marital status at the third follow-up, total energy intake, smoking status, alcohol consumption, physical activity level, BMI, sleep duration, weekly supplement use, baseline medical history of hypertension, cardiovascular diseases, and diabetes, and instrumental limitations, self-rated health and social activity at the third follow-up. ^a^ All *P*_trend_ < 0.05.

After accounting for potential confounders, compared to those in the lowest quartiles, those in the highest quartiles for intake of vitamin C, carotenoids, and flavonoids, but not of vitamin E, had significantly reduced odds of late-life depressive symptoms (Figure 2). Specifically, the inverse associations between antioxidant intake and late-life depressive symptoms were observed for the intakes of all carotenoids except for lutein and for all flavonoids except for anthocyanins.

## 4. Discussion

To the best of our knowledge, our study provided the first prospective population-based evidence of a significant association between dietary TAC in midlife and a likelihood of depressive symptoms at older age. In this cohort study among middle-aged and older Chinese adults living in Singapore, we found that higher antioxidant capacity of midlife diet, measured by CDAI and VCEAC indices, was significantly associated with a lower likelihood of depressive symptoms in late life. Analyses of individual components of the dietary TAC showed that dietary intakes of vitamin C, total carotenoids and total flavonoids were associated with a reduced likelihood of late-life depressive symptoms. 

Hitherto, several epidemiological studies have investigated the association between individual antioxidant nutrients and late-life depressive symptomology, although most of them were cross-sectional studies with relatively small sample sizes, and the results have remained inconsistent [29,30,31,32,33,34]. A cross-sectional study among 1634 Japanese adults aged ≥ 65 years indicated that dietary intakes of β-carotene, cryptoxanthin, α-tocopherol, and vitamin C were significantly lower in participants with depressive symptoms compared to those without depressive symptoms [29]. In a case-control study among 144 depressed patients and 134 comparison participants who were aged ≥ 60 years, the cases had significantly lower intakes of dietary vitamin C and cryptoxanthin, while no significant difference was observed for the intakes of dietary vitamin E and other carotenoids [30]. In a cohort study involving 781 men with a mean age of 81.4 years from the Concord Health and Ageing in Men Project, the authors assessed dietary intakes of vitamins A, C, E and zinc at baseline and after 3 years of follow-up; they found that while those in the lowest quartile of dietary vitamin E were 1.18 times more likely to have incident depressive symptoms compared to those in the highest quartile, no association was observed between dietary vitamin C and depressive symptoms [31]. Regarding flavonoids, the Nurses’ Health Study in the USA analyzed a sample of 41,920 women aged ≥ 65 years over an average of 10 years’ follow-up and found that a higher intake of flavonoids was associated with a lower risk of late-life depression [32]. However, findings from randomized controlled trials investigating the effect of flavonoid supplementation on late-life depression were mixed, as both beneficial and null effects have been observed in different trials [33,34]. The discrepant results for individual antioxidant nutrients mainly arose from differences in study design, the specific targeted populations, and variations in antioxidant intake across diverse geographic regions and socioeconomic groups.

Based on these mixed findings, it has been suggested that isolated antioxidants may not fully reflect the collective antioxidant capacity present in our usual diet, thus leading to an underestimation of combined protective effects from a variety of antioxidant nutrients present in the diet. As such, the concept of dietary TAC, which considers the synergistic interactions of the antioxidants present in the food matrix, has been proposed to measure the complex network of food-based antioxidants [35]. In our study, the two TAC indices, namely CDAI and VCEAC, have been associated with lower serum inflammatory markers in population-based observational studies [36,37]. Our results concurred with findings from previous cross-sectional studies that focused on peri- and postmenopausal women who were at risk of menopausal-associated depression [38]. In a cross-sectional study among 265 women with diabetes at a mean age of 60 years, women with depression had significantly lower dietary TAC in their diet (indicated by FRAP and ORAC) compared to those without depression [16]. Similarly, another cross-sectional study among 175 postmenopausal women also showed that dietary TAC (indicated by ORAC) was inversely associated with depression [15]. However, the cross-sectional design of these previous studies raised the possibility of reverse causality since individuals with depression tend to exhibit altered food preferences and heterogeneity in appetite [39]. These changes can manifest as either decreased appetite or cravings for energy-dense foods high in carbohydrates and fat [40,41], both of which may potentially lead to inadequate intake of dietary antioxidants. Our research addressed these limitations by employing a prospective design within a general population sample, which provided more convincing evidence with regard to the long-term relationship between antioxidant intake from a midlife diet and the subsequent likelihood of depression in late life.

The biological plausibility of our findings is supported by a growing body of evidence that has shown elevated oxidative stress and neuroinflammation could be the main contributors to the development of aging-related depression [5,6,42], and endogenous supplies for the antioxidant defense systems in human decline with aging [43]. Dietary antioxidants can contribute to the exogenous supplies of antioxidants that act as scavengers of excessive ROS and thus counteract neurological damage from oxidative stress by mitigating inflammatory status in the brain [11]. In addition, these dietary antioxidants have also been found to beneficially alter gut-redox potential and module expression of immunoglobulin A, a factor important for bacterial colonization in the gut [44], which could consequently impact mood regulation and depressive symptoms through the gut-brain axis [45]. Furthermore, antioxidant nutrients play a role in regulating neurotransmitter production and modulating signaling pathways, which are critical for maintaining neuron survival and synaptic plasticity [46].

The strengths of this novel study include the large sample size, prospective design with a long follow-up period, and a comprehensive collection of confounding factors that enabled us to rigorously investigate the prospective relationship between midlife dietary antioxidant capacity and late-life depressive symptoms. We comprehensively assessed dietary intake of nutrients using a validated FFQ and constructed two dietary antioxidant capacity indices based on well-established methods. However, several limitations also need to be noted. First, dietary nutrients were only assessed at baseline, which could not capture participants’ subsequent changes in dietary habits. Nevertheless, previous prospective studies have shown that dietary patterns in the general population tend to remain stable after midlife [47] despite expected changes due to medical conditions or evolving health perceptions. In this regard, we performed a sensitivity analysis by excluding participants with baseline chronic diseases, such as diabetes, hypertension and cardiovascular diseases, and the results did not change materially. Second, selection bias was possible given that we only successfully re-interviewed a proportion of those enrolled at baseline. To address this, we performed an inverse probability weighted model to partially handle the bias caused by censoring or missing data and found that the results remained largely consistent. Third, depressive symptoms were self-reported and evaluated using a questionnaire instrument (GDS-15) rather than through clinical diagnostic interviews, which could result in misclassification of depression. Nonetheless, GDS-15 is a well-validated screening tool that has been used widely in population-based studies of older adults [48]. Fourth, we did not measure depressive symptoms at baseline interviews, which were conducted, on average, about 19.6 years before our first measurement of depressive symptoms at follow-up 3 visits. Nonetheless, to mitigate the possibility of reverse causality, we excluded those who were already 65 years and older at baseline or those with self-reported clinical depression at follow-up 3. Fifth, we did not collect blood samples at the measurement of diet habits to validate self-report of intake. As blood concentration of antioxidants represents a dynamic equilibrium of dietary intake, metabolism and individual redox state [11], lack of information on circulating levels of antioxidants may limit our ability to examine how dietary intake contributes to in vivo antioxidant defenses and their association with late-life depressive symptoms. Sixth, as we did not collect information on antidepressant and other substance use, we were unable to account for their potential mediating or modifying effects on the association between antioxidant intake and late-life depressive symptoms. Finally, despite the adjustment for various potential confounders, bias could not be precluded completely due to residual confounding, and caution should be taken when generalizing our findings to other populations.

## 5. Conclusions

In conclusion, our study showed that a high level of dietary antioxidant capacity in midlife was associated with a lower likelihood of depressive symptoms in late life and provided evidence for the recommendation of an antioxidant-rich diet for the prevention of depression in aging. Future studies with repeated dietary assessments are still needed to confirm these associations and to elucidate the specific mechanisms through which dietary antioxidants may protect against aging-related depression.

## Figures and Tables

**Table 1:** Characteristics of participants by depressed status measured at follow-up 3.

Characteristics	Total	Non-Depressed	Depressed
n of participants	13,712	10,539	3173
Age at baseline, y	52.4 ± 5.9	52.0 ± 5.7	53.7 ± 6.3 *
Age at follow-up 3, y	72.5 ± 6.1	72.1 ± 5.9	74.1 ± 6.4 *
Women, %	7969 (58.1)	5974 (56.7)	1995 (62.9) *
Married at follow-up 3, %	9674 (70.6)	7640 (72.5)	2034 (64.1) *
Dialect group, %			
Cantonese	6721(49.0)	5293(50.2)	1428(45.0) *
Hokkien	6991(51.0)	5246(49.8)	1745(55.0)
Education, %			
No formal education	2470 (18.0)	1576 (15.0)	894 (28.1) *
Primary school	6237 (45.5)	4648 (44.1)	1589 (50.1)
Secondary school or higher	5005 (36.5)	4315 (40.9)	690 (21.8)
BMI, kg/m^2^	23.1 ± 3.2	23.0 ± 3.1	23.4 ± 3.4 *
Daily energy intake, kcal	1595.3 ± 517.6	1602.8 ± 519.8	1570.5 ± 509.5 *
6–8 h/day of sleep, %	11943 (87.1)	9268 (87.9)	2675 (84.3) *
Current smoker, %	1795 (13.1)	1263 (12.0)	532 (16.8) *
Daily Drinker, %	368 (2.7)	276 (2.6)	92 (2.9)
Physical inactive ^a^, %	8627 (62.9)	6423 (61.0)	2204 (69.5) *
Weekly supplement use, %	1052 (7.7)	873 (8.3)	179 (5.6) *
Baseline hypertension, %	2553 (18.6)	1852 (17.6)	701 (22.1) *
Baseline cardiovascular diseases, %	305 (2.2)	186 (1.8)	119 (3.8) *
Baseline diabetes, %	615 (4.5)	423 (4.0)	192 (6.1) *
**Aging-related factors at follow-up 3**, %			
At least one limitation in IADL	3147 (23.0)	2002 (19.0)	1145 (36.1) *
Fair or poor self-rated health	6969 (50.8)	4765 (45.2)	2204 (69.5) *
No social activity	6275 (45.8)	4537 (43.1)	1738 (54.8) *
**Dietary total antioxidant capacity and its individual components**			
CDAI ^b^	−0.3 (−1.7–1.7)	−0.2 (−1.6–1.9)	−0.7 (−2.0–1.1) *
VCEAC ^b^	258.8 (152.6–497.6)	269.2 (158.9–520.9)	227.2 (135.5–427.1) *
Vitamin C, mg/d	84.1 (56.5–125.5)	87.5 (58.3–130.0)	75.3 (51.4–110.8) *
Vitamin E, mg/d	6.3 (5.0–7.6)	6.3 (5.0–7.6)	6.2 (4.9–7.5) *
Total carotenoids, mcg/d	5484.4 (3998.6–7459.1)	5572.6 (4062.7–7590.1)	5246.9 (3835.8–7035.0) *
α-carotene	196.9 (108.7–339.6)	202.5 (111.4–347.9)	178.8 (99.9–313.1) *
β-carotene	2049.1 (1456.5–2898.8)	2077.1 (1477.3–2941.6)	1967.8 (1394.2–2744.3) *
β-cryptoxanthin	180.3 (90.6–322.5)	188.3 (96.7–334.3)	156.3 (76.9–278.5) *
Lycopene	866.1 (452.0–1488.7)	888.8 (465.6–1524.3)	797.3 (404.3–1353.9) *
Lutein	1788.1 (1305.9–2450.8)	1800.7 (1320.3–2461.2)	1750.8 (1259.8–2416.9) *
Total flavonoids, mg/d	101.2 (48.3–271.1)	105.3 (51.2–282.5)	89.1 (42.6–230.2) *
Anthocyanins	2.0 (0.9–3.5)	2.0 (1.0–3.6)	1.8 (0.9–3.2) *
Flavan-3-ols	60.4 (18.6–229.3)	62.6 (19.8–237.6)	54.2 (15.4–185.8) *
Flavanones	18.0 (6.2–33.2)	19.3 (6.8–35.2)	14.1 (4.9–28.7) *
Flavones	0.9 (0.5–1.5)	0.9 (0.5–1.6)	0.7 (0.4–1.3) *
Flavonols	8.2 (5.5–13.7)	8.4 (5.7–14.1)	7.8 (5.2–12.5) *

Abbreviations: CDAI, Component Dietary Antioxidant Index; VCEAC, Vitamin C Equivalent Antioxidant Capacity; BMI, body mass index; IADL, Lawton instrumental activities of daily living scale. ^a^ Physically inactive was defined as having any weekly moderate activity, vigorous activity, or strenuous sports lasting less than 30 min. ^b^ The CDAI with negative values was computed from standardized antioxidant intake and principal component analysis and had no unit; the unit for VCEAC was vitamin C equivalents per 100 g. Values are means ± SDs or medians (interquartile ranges) or numbers (percentages) as appropriate. The chi-square test was used for the comparison of categorical variables, and Student’s *t*-test or the Mann–Whitney U test was used for the comparison of continuous variables as appropriate. * *p* < 0.05.

**Table 2:** Characteristics of participants by extreme quartiles for dietary total antioxidant capacity.

Characteristics		CDAI		VCEAC	
	Total	Q1	Q4	Q1	Q4
n of participants	13,712	3428	3428	3428	3428
Age at baseline, y	52.4 ± 5.9	52.7 ± 6.1	52.1 ± 5.8 *	52.4 ± 6.0	52.4 ± 5.9
Age at follow-up 3, y	72.5 ± 6.1	72.9 ± 6.2	71.9 ± 5.9 *	72.5 ± 6.1	72.3 ± 6.1 *
Women, %	7969 (58.1)	1529 (44.6)	2098 (61.2) *	1693 (49.4)	1786 (52.1) *
Married at follow-up 3, %	9674 (70.6)	2458 (71.7)	2460 (71.8)	2488 (72.6)	2496 (72.8)
Dialect group					
Cantonese	6721 (49.0)	1508 (44.0)	1955 (57.0) *	1591 (46.4)	1886 (55.0) *
Hokkien	6991 (51.00)	1920 (56.0)	1473 (43.0)	1837 (53.6)	1542 (45.0)
Education, %					
No formal education	2470 (18.0)	713 (20.8)	400 (11.7) *	705 (20.6)	380 (11.1) *
Primary school	6237 (45.5)	1691 (49.3)	1390 (40.6)	1692 (49.4)	1504 (43.9)
Secondary school or higher	5005 (36.5)	1024 (29.9)	1638 (47.8)	1031 (30.1)	1544 (45.0)
BMI, kg/m^2^	23.1 ± 3.2	22.9 ± 3.2	23.3 ± 3.2 *	22.8 ± 3.1	23.4 ± 3.1 *
Daily energy intake	1595.3 ± 517.6	1733.3 ± 544.5	1650.7 ± 508.5 *	1861.5 ± 511.3	1627.3 ± 512.4 *
6–8 h/day of sleep, %	11943 (87.1)	2969 (86.6)	2964 (86.5)	2966 (86.5)	3003 (87.6)
Current smoker, %	1795 (13.1)	778 (22.7)	286 (8.3) *	677 (19.8)	417 (12.2) *
Daily Drinker, %	368 (2.7)	169 (4.9)	55 (1.6) *	139 (4.1)	85 (2.5) *
Physical inactive ^a^, %	8627 (62.9)	2233 (65.1)	1900 (55.4) *	2263 (66.0)	1901 (55.5) *
Weekly supplement use, %	1052 (7.7)	46 (1.3)	763 (22.3) *	75 (2.2)	584 (17.0) *
Baseline hypertension, %	2553 (18.6)	549 (16.0)	712 (20.8) *	531 (15.5)	744 (21.7)
Baseline cardiovascular diseases, %	305 (2.2)	75 (2.2)	80 (2.3)	67 (2.0)	88 (2.6)
Baseline diabetes, %	615 (4.5)	150 (4.4)	143 (4.2)	130 (3.8)	176 (5.1) *
**Aging-related factors at follow-up 3**, %					
Depressed defined as GDS ≥ 5	3173 (23.1)	956 (27.9)	603 (17.6) *	939 (27.4)	650 (19.0) *
At least one limitation in IADL	3147 (23.0)	950 (27.7)	596 (17.4) *	892 (26.0)	676 (19.7) *
Fair or poor self-rated health	6969 (50.8)	1830 (53.4)	1536 (44.8) *	1806 (52.7)	1636 (47.7) *
No social activity	6275 (45.8)	1728 (50.4)	1386 (40.4) *	1728 (50.4)	1417 (41.3) *

^a^ Physically inactive was defined as having any weekly moderate activity, vigorous activity, or strenuous sports lasting less than 30 min. Values are means ± SDs or medians (interquartile ranges) or numbers (percentages) as appropriate. The chi-square test and analysis of variance were used for the comparison of categorical and continuous variables, respectively. * *p* < 0.05.

**Table 3:** Association between dietary total antioxidant capacity in midlife and odds of depressive symptoms in late life (n = 13,712).

	Quartile of Dietary Total Antioxidant Capacity				
	Q1	Q2	Q3	Q4	*P*_trend_ ^a^
**CDAI**					
Case/n	956/3428	849/3428	765/3428	603/3428	
Value of range	−7.9 to −1.7	−1.7 to −0.3	−0.3 to 1.7	1.7 to 35.2	
Model 1	1.00	0.85 (0.76–0.95)	0.74 (0.67–0.83)	0.55 (0.49–0.62)	<0.01
Model 2	1.00	0.87 (0.78–0.98)	0.81 (0.72–0.91)	0.67 (0.59–0.76)	<0.01
Model 3	1.00	0.88 (0.78–0.98)	0.85 (0.75–0.95)	0.73 (0.64–0.83)	<0.01
**VCEAC**					
Case/n	939/3428	852/3428	732/3428	650/3428	
Value of range	−270.9 to 152.6	152.6 to 258.8	258.8 to 497.3	497.8 to 6192.5	
Model 1	1.00	0.88 (0.79–0.98)	0.72 (0.64–0.80)	0.62 (0.55–0.70)	<0.01
Model 2	1.00	0.86 (0.77–0.97)	0.79 (0.70–0.89)	0.72 (0.63–0.81)	<0.01
Model 3	1.00	0.88 (0.78–1.00)	0.83 (0.74–0.94)	0.77 (0.68–0.87)	<0.01

Model 1 was unadjusted; model 2 was adjusted for age and marital status at the third follow-up, sex, level of education, dialect group, total energy intake, smoking status, alcohol consumption, physical activity level, BMI, sleep duration, weekly supplement use, baseline medical history of hypertension, diabetes, cardiovascular diseases; model 3 additionally adjusted for instrumental limitations, self-rated health and social activity at the third follow-up. ^a^ Linear trend was assessed by including the median values of each respective quartile as continuous variables in models.

## Data Availability

The dataset used in this study is available from the corresponding author upon request.

## Supplementary Materials

The following supporting information can be downloaded at https://www.mdpi.com/article/10.3390/antiox13050576/s1, References [49,50,51] are cited in the supplementary materials. Table S1: Association between the dietary total antioxidant capacity in midlife and odds of depressive symptoms in late life: stratified by age at GDS assessment, sex, BMI, smoking status, and history of chronic diseases at baseline; Table S2: IPW models for the association between the dietary total antioxidant capacity in midlife and odds of depressive symptoms in late life; Table S3: Association between dietary total antioxidant capacity in midlife and odds of depressive symptoms in late life excluding participants with chronic diseases at baseline; Table S4: Association between dietary total antioxidant capacity in midlife and odds of depressive symptoms in late life excluding participants aged ≥ 65 years at baseline.
