# Supplementary material for: Association between Dietary Antioxidant Capacity in Midlife and Depressive Symptoms in Late Life: The Singapore Chinese Health Study
Source: Antioxidants (Basel). 2024 May 8;13(5):576. doi: 10.3390/antiox13050576 (PMC11118857; doi:10.3390/antiox13050576)
Supplement: Supplementary file 1 [file antioxidants-13-00576-s001.zip › antioxidants-2962600-supplementary.pdf]

Association between dietary antioxidant capacity in midlife and depressive symptoms in late life: the Singapore Chinese Health Study

Description of supplemental materials:

Supplemental methods

Supplement Table S1 Association between the dietary total antioxidant capacity in midlife and odds of depressive symptoms in late life: stratified by age at GDS assessment, sex, BMI, smoking status, and history of chronic diseases at baseline.

Supplement Table S2 IPW models for the association between the dietary total antioxidant capacity in midlife and odds of depressive symptoms in late life (n=13,712).

Supplement Table S3 Association between dietary total antioxidant capacity in midlife and odds of depressive symptoms in late life excluding participants with chronic diseases at baseline (n= 10,634).

Supplement Table S4 Association between dietary total antioxidant capacity in midlife and odds of depressive symptoms in late life after excluding participants aged ≥65 years at baseline (n= 13,177).

Supplemental methods.

Construction of the inverse probability weighted regression model.

The inverse probability weighted marginal structural model was introduced by Robins and colleagues [40-51]. Taking the analysis for the Vitamin C Equivalent Antioxidant Capacity (VCEAC) as an example, we first specified the binary logit models to get the stabilized weights for censoring (*sw–c*), with the predicted probability of being non-censored given VCEAC in the numerator, and the predicted probability of being non-censored given VCEAC with other potential confounders for depressive symptoms (the same as listed in the final model of the logistic regression analyses) in the denominator.

$$sw-c = \frac{pr(cencering = 0|VCEAC)}{pr(cencering = 0|VCEAC, covariates *)}$$

Second, we specified the ordinal logit models to get the stabilized weights for confounding (*sw–e*), with the probability of being in the observed quartile group of VCEAC in the numerator and the probability of being in the observed quartile group of VCEAC given the potential confounders for depressive symptoms (the same as listed in the final logistic model) in the denominator.

*covariates included age, sex, dialect groups, educational level, marital status, physical activities, body mass index, smoking status, alcohol consumption, sleep duration, total daily energy intake, weekly supplement use, and physician-diagnosed history of diabetes, hypertension, cardiovascular disease.

$$sw-e = \frac{pr(VCEAC)}{pr(VCEAC|covariates *)}$$

The continuous variables (age and daily energy intake) were included in the models with 5-knot natural cubic splines with knots placed at the fifth, 25th, 50th, 75th, 95th percentiles. The final inverse probability weights were computed as the product of the stabilized weights for censoring times the stabilized weights for confounding adjustment and then truncated at the first and 99th percentiles, which was recommended to reduce the magnitude in the 1/minimum and maximum weights. We utilized the inverse probability weights and performed the weighted logistic regression analyses using the SAS program “Proc Genmod” to obtain the estimates [51].

Supplement Table S1. Association between the dietary total antioxidant capacity in midlife and odds of depressive symptoms in late life: stratified by age at GDS assessment, sex, BMI, smoking status, and history of chronic diseases at baseline.

		Quartile of dietary total antioxidant capacity				p_{trend}[a]	$p_{interaction}$[b]
	Cases/n	Q1	Q2	Q3	Q4		
**Stratified by age at GDS assessment**							
**CDAI**							
<70 years	970/5638	1.00	0.91 (0.74-1.11)	0.86 (0.70-1.06)	0.72 (0.57-0.89)	<0.01	0.92
≥70 years	2203/8074	1.00	0.86 (0.74-0.99)	0.83 (0.72-0.96)	0.72 (0.62-0.85)	<0.01	
**VCEAC**							
<70 years	970/5638	1.00	0.88 (0.71-1.08)	0.76 (0.62-0.94)	0.79 (0.64-0.97)	0.07	0.51
≥70 years	2203/8074	1.00	0.89 (0.76-1.03)	0.88 (0.75-1.03)	0.76 (0.65-0.88)	<0.01	
**Stratified by sex**							
**CDAI**							
Men	1178/5743	1.00	0.89 (0.74-1.07)	0.93 (0.77-1.13)	0.75 (0.61-0.92)	0.01	0.58
Women	1995/7969	1.00	0.85 (0.73-0.99)	0.79 (0.67-0.92)	0.69 (0.59-0.82)	<0.01	
**VCEAC**							
Men	1178/5743	1.00	0.85 (0.69-1.04)	0.84 (0.69-1.02)	0.76 (0.63-0.91)	0.01	0.94
Women	1995/7969	1.00	0.91 (0.78-1.07)	0.83 (0.71-0.98)	0.77 (0.64-0.91)	<0.01	
**Stratified by baseline BMI**							
**CDAI**							
<23kg/m^2	1430/6789	1.00	0.92 (0.78-1.08)	0.89 (0.75-1.06)	0.73 (0.60-0.88)	<0.01	0.81
≥23kg/m^2	1743/6923	1.00	0.83 (0.71-0.98)	0.80 (0.68-0.94)	0.72 (0.61-0.86)	<0.01	
**VCEAC**							0.87
<23kg/m^2	1430/6789	1.00	0.84 (0.70-0.99)	0.81 (0.68-0.97)	0.76 (0.63-0.92)	0.02	
≥23kg/m^2	1743/6923	1.00	0.93 (0.79-1.11)	0.86 (0.72-1.02)	0.78 (0.66-0.93)	0.01	
**Stratified by smoking status**							
**CDAI**							0.50
Non-smoker	2346/10664	1.00	0.84 (0.73-0.97)	0.80 (0.69-0.92)	0.70 (0.60-0.82)	<0.01	
Ever smoker	827/3048	1.00	0.94 (0.76-1.17)	0.95 (0.75-1.20)	0.75 (0.58-0.98)	0.04	
**VCEAC**							
Non-smoker	2346/10664	1.00	0.85 (0.74-0.97)	0.84 (0.72-0.97)	0.73 (0.63-0.85)	<0.01	0.24

Ever smoker	827/3048	1.00	0.99 (0.78-1.27)	0.76 (0.59-0.97)	0.82 (0.65-1.03)	0.10	
**Stratified by history of chronic diseases at baseline [c]**							
CDAI							
No	2305/10634	1.00	0.91 (0.80-1.04)	0.85 (0.74-0.97)	0.71 (0.61-0.83)	<0.01	0.23
Yes	868/3078	1.00	0.75 (0.59-0.96)	0.84 (0.66-1.06)	0.75 (0.58-0.96)	0.08	
VCEAC							
No	2305/10634	1.00	0.88 (0.77-1.01)	0.85 (0.74-0.98)	0.77 (0.66-0.89)	<0.01	0.69
Yes	868/3078	1.00	0.87 (0.67-1.12)	0.78 (0.61-1.01)	0.75 (0.58-0.96)	0.04	

Models were adjusted for age at the third follow-up, sex, dialect group, level of education, marital status at the third follow-up, total energy intake, smoking status, alcohol consumption, physical activity level, BMI, sleep duration, weekly supplement use, baseline medical history of hypertension, cardiovascular diseases, and diabetes, and instrumental limitations, self-rated health and social activity at the third follow-up. Stratified factors were excluded in respective models.

[a] Linear trend was tested by treating the median values of quartiles as a continuous variable.

[b] Significance of the interaction was tested by including a cross-product term between dietary total antioxidant capacity and the stratification factors in the model.

[c] Chronic diseases include hypertension, cardiovascular diseases, and diabetes

Supplement Table S2. IPW models for the association between the dietary total antioxidant capacity in midlife and odds of depressive symptoms in late life (n=13,712).

	Median	Cases/n	OR (95% CI)
CDAI			
Q1	-2.55	956/3428	1.00
Q2	-1.03	849/3428	0.91 (0.76-1.08)
Q3	0.56	765/3428	0.91 (0.76-1.08)
Q4	3.77	603/3428	0.80 (0.66-0.97)
p for trend [b]			0.03
VCEAC			
Q1	90.38	939/3428	1.00
Q2	202.55	852/3428	0.96 (0.80-1.15)
Q3	342.08	732/3428	0.89 (0.74-1.08)
Q4	936.06	650/3428	0.79 (0.66-0.95)
p for trend [b]			0.01

Abbreviations: IPW, inverse probability weighting; CDAI, Component Dietary Antioxidant Index; VCEAC, Vitamin C Equivalent Antioxidant Capacity.

Model was adjusted for age and marital status at the third follow-up, sex, level of education, dialect group, total energy intake, smoking status, alcohol consumption, physical activity level, body mass index, sleep duration, weekly supplement use, baseline medical history of hypertension, diabetes, cardiovascular diseases, instrumental limitations, self-rated health and social activity at the third follow-up.

[b] Liner trend was assessed by including median values of each respective quartile as continuous variables in models.

Supplement Table S3. Association between dietary total antioxidant capacity in midlife and odds of depressive symptoms in late life excluding participants with chronic diseases at baseline (n= 10,634).

	Median	Cases/n	OR (95% CI)
CDAI			
Q1	-2.60	700/2658	1.00
Q2	-1.07	640/2659	0.91 (0.79-1.04)
Q3	0.51	541/2659	0.84 (0.73-0.97)
Q4	3.64	424/2658	0.72 (0.62-0.84)
p for trend [a]			<0.01
VCEAC			
Q1	87.66	680/2658	1.00
Q2	197.28	633/2659	0.92 (0.80-1.06)
Q3	332.01	529/2659	0.85 (0.74-0.98)
Q4	903.76	463/2658	0.78 (0.68-0.91)
p for trend [a]			0.01

Abbreviations: CDAI, Component Dietary Antioxidant Index; VCEAC, Vitamin C Equivalent Antioxidant Capacity.

Model was adjusted for age and marital status at the third follow-up, sex, level of education, dialect group, total energy intake, smoking status, alcohol consumption, physical activity level, body mass index, sleep duration, weekly supplement use, and instrumental limitations, self-rated health and social activity at the third follow-up.

[a] Liner trend was assessed by including median values of each respective quartile as continuous variables in models.

Supplement Table S4. Association between dietary total antioxidant capacity in midlife and odds of depressive symptoms in late life excluding participants aged ≥65 years at baseline (n= 13,177).

	Median	Cases/n	OR (95% CI)
CDAI			
Q1	-2.55	896/3294	1.00
Q2	-1.02	804/3294	0.89 (0.79-1.00)
Q3	0.58	717/3295	0.85 (0.75-0.96)
Q4	3.78	556/3294	0.73 (0.63-0.83)
p for trend [a]			<0.01
VCEAC			
Q1	89.51	885/3294	1.00
Q2	202.87	800/3294	0.88 (0.77-0.99)
Q3	341.86	684/3295	0.84 (0.74-0.95)
Q4	932.06	604/3294	0.77 (0.68-0.88)
p for trend [a]			0.01

Abbreviations: CDAI, Component Dietary Antioxidant Index; VCEAC, Vitamin C Equivalent Antioxidant Capacity.

Model was adjusted for age and marital status at the third follow-up, sex, level of education, dialect group, total energy intake, smoking status, alcohol consumption, physical activity level, BMI, sleep duration, weekly supplement use, baseline medical history of hypertension, diabetes, cardiovascular diseases, and instrumental limitations, self-rated health and social activity at the third follow-up.

[a] Linear trend was assessed by including median values of each respective quartile as continuous variables in models.
